# Gut Microbiota as Regulators of Th17/Treg Balance in Patients With Myasthenia Gravis

**DOI:** 10.3389/fimmu.2021.803101

**Published:** 2021-12-23

**Authors:** Pan Chen, Xiangqi Tang

**Affiliations:** Department of Neurology, The Second Xiangya Hospital, Central South University, Changsha, China

**Keywords:** myasthenia gravis, Th17, Treg, gut microbiota, short-chain fatty acids

## Abstract

Myasthenia gravis (MG) is an acquired neurological autoimmune disorder characterized by dysfunctional transmission at the neuromuscular junction, with its etiology associated with genetic and environmental factors. Anti-inflammatory regulatory T cells (Tregs) and pro-inflammatory T helper 17 (Th17) cells functionally antagonize each other, and the immune imbalance between them contributes to the pathogenesis of MG. Among the numerous factors influencing the balance of Th17/Treg cells, the gut microbiota have received attention from scholars. Gut microbial dysbiosis and altered microbial metabolites have been seen in patients with MG. Therefore, correcting Th17/Treg imbalances may be a novel therapeutic approach to MG by modifying the gut microbiota. In this review, we initially review the association between Treg/Th17 and the occurrence of MG and subsequently focus on recent findings on alterations of gut microbiota and microbial metabolites in patients with MG. We also explore the effects of gut microbiota on Th17/Treg balance in patients with MG, which may provide a new direction for the prevention and treatment of this disease.

## Introduction

Myasthenia gravis (MG) is an autoantibody-mediated neurological disorder caused by transmission defects at the neuromuscular junction (1). Pathogenic antibodies that can be detected using current technological methods mainly include antibodies against acetylcholine receptors (AChR), muscle-specific kinase (MuSK), and lipoprotein receptor-related protein 4 (LRP4) (2). The antibodies induce fatigue and weakness of the eye muscles or skeletal muscles, which are the main clinical hallmarks (3, 4). Patients with MG whose weakness is limited to the extraocular muscles are referred to as patients with oculomotor myasthenia gravis (OMG). Once fatigue spreads to the skeletal muscles, the disease can progress to generalized myasthenia gravis (GMG). Depending on whether patients are over 50 years of age at onset, MG can be divided into early-onset MG (EOMG) and late-onset MG (LOMG) (4). Myasthenia gravis has an annual worldwide incidence of 8–10 per 1 million people per year, and the total prevalence of MG has been reported to be 150–250 per 1 million people (5). Genetic factors play a crucial role in the induction of myasthenia gravis. MG has a concordance rate of 35.5% in monozygotic twins and 4-5% in heterozygous twins (6). As *reported*,some genes correlates strongly with *the onset of MG,such as* HLA-DQ5 and polymorphisms of CTLA-4 (4, 7). *Based on these findings, genetic susceptibility may contribute to the development of MG.*With a low rate of spontaneous remission, most patients require long-term immunosuppressive agents to achieve minimal manifestation status or improvement after MG intervention (2). Monoclonal antibodies targeting specific molecules have become a research hotspot in recent years; however, they are not yet widely applied in clinical practice, and finding treatments to prevent or cure this disorder is a major challenge in this field that requires an in-depth understanding of disease pathogenesis.

Myasthenia gravis is an acquired autoimmune disease with complex pathogenesis, mediated by humoral and cellular immunity and implicated by complement. The thymus is affected in most AChR-MG patients, with thymic hyperplasia being the most common (8).The formation of germinal centers in thymus is a typical pathological change of MG patients (9).Take early-onset AChR-MG for example, in response to AChR stimulation, the thymic germinal centers overexpress pro-inflammatory cytokines and thymic epithelial cells, presenting AChR subunits to autoreactive CD4+ T cells, thereby upregulating IL-4 and IL-6 and stimulating B cell proliferation to promote the production of anti-AChR antibodies (10).Meanwhile, the titers of IFN-γ and IL-17 are increased, implying the involvement of T helper (Th)1 and Th17 cells in MG pathogenesis (11). Moreover, the ability of Treg to suppress T cell responses are also greatly impaired (11). Tregs are critical for maintaining immune tolerance and immune homeostasis (12). Th17 cells play an essential role in mediating chronic inflammation in autoimmune diseases (13), and multiple studies have shown abnormalities in the function or expression levels of Treg and Th17 in animal models or patients with MG (14–20). Moreover, antibody titer tends to correlate with disease severity, indicating a possible involvement of a Treg/Th17 imbalance in MG disease pathogenesis. Rebuilding Thl7/Treg balance has a promising application for the biological target therapy of MG.

Gut microbiota can influence various human physiological activities in many aspects, including nutrient synthesis, immune response modulation, and defense against pathogen invasion (21–23). The gut microbiome is a dynamically balanced unity of microorganisms. Although the mechanisms are still unclear, the immunomodulatory effects of gut microbiota are mostly realized through the Thl7/Treg axis (24, 25). Gut microbiota dysbiosis may lead to increased permeability of the intestinal mucosal barrier, infection, and excessive inflammatory response, as well as an imbalance of T cell subsets, which has been described in asthma (26), rheumatoid arthritis (27), inflammatory bowel disease (28), multiple sclerosis (29), and MG (30), among others. Accordingly, regulating the immune imbalance of Thl7/Treg cells and inducing the reconstruction of immune tolerance may become a potential treatment modality for autoimmune diseases, such as MG. Studies have revealed *that* environmental factors, especially dietary factors, can *alter* the gut microbiota *composition* and its *metabolic activity*, which produce adverse implications for human health (31). For instance, alterations in the gut microbiota caused by a Western high-fat diet have recently been recognized as a major contributing factor to the *colorectal cancer* epidemic (32)*. High*-sucrose-*high*-*fat dietary patterns* were supposed to participate *in* the asthma *pathogenesis through* intestinal bacteria (33, 34). Currently, there is not related research on whether dietary factors are involved in the pathology of MG. This review paper covers three main aspects. First, we introduce the association between Treg/Th17 imbalances and MG occurrence. Second, we summarize recent findings regarding varied gut microbiota and its metabolites in patients with MG and also explore the effects of gut microbiota on Th17/Treg in these patients. Finally, we provide new insights on MG therapy regarding the rebuilding of Th17/Treg balance by modifying the gut microbiota.

## Significance of Th17/Treg Imbalances in MG Progression

Th17 cells synthesize and release various inflammatory cytokines, such as interleukin (IL)-17 and IL-22, activate neutrophils, and infiltrate into lesions, thus amplifying inflammatory effect. The key transcription factor, retinoic acid-related orphan receptor (RORγt), mediates the differentiation of Th17 cells and biosynthesis of Th17 cytokines (35–37). Evidence from previous studies has shown that Th17 cells promote inflammatory responses and mediate tissue damage. The pro-inflammatory effects of Th17 cells are mainly attributed to their secretion of IL-17, and the corresponding mechanisms involves the recruitment of neutrophils, enhancement of B cell function, activation of innate immune cells, and induction release of pro-inflammatory cytokines, such as tumor necrosis factor alpha, granulocyte-macrophage colony-stimulating factor, and IL-1β (38, 39). Various studies have found that Thl7 cells and their main secreted cytokine IL-17 is related inextricably to MG pathogenesis. Schaffert et al. observed less myasthenia symptoms and significant reductions in anti-AChR antibodies in IL-17(knock-out) mice, concluding that the process of antibody production and loss of B-cell tolerance was regulated by IL-17 and consequently confirming the involvement of Th17 cells in MG autoimmunity (14). A study in 2017 emphasized that IL-17 independent pathways drove the autoimmune response related to experimental autoimmune myasthenia gravis (EAMG) development (15). Moreover, serum IL-17 levels have shown a positive correlation to the degree of quantitative MG score and anti-AChR antibody titers, heralding a severer disease course (16). Antibody production in patients with MG is affected by Th1/Th2 cytokine balances, which is regulated by Th17 cells (40). Accordingly, Th17 cells and their released cytokines are linked to the induction of anti-AChR antibody-mediated autoimmunity at the neuromuscular junction.

Tregs could inhibit the function of other effector T cells and antigen-presenting cells, suppressing immune responses (41). Tregs release multiple inhibitory cytokines, such as transforming growth factor-beta (TGF-β) and IL-10 (42). The immunomodulatory property of Tregs is controlled by the expression of forkhead box protein 3 (FoxP3) (43). The number of CD4+CD25+FoxP3+Tregs in peripheral blood is decreased during the active stage of anti-AChR antibody-positive MG and becomes higher with immunotherapy, implying that decreased number of Tregs may lead to immune disorders (44). The number of Tregs seems to be influenced by disease involvement. For instance, the proportion of Tregs of generalized anti-AChR antibody positive MG without thymoma group is remarkably lower than those of ocular MG, as could be seen in the study by Hu et al. (17). However, research has demonstrated that the relative number of Tregs presented no obvious changes when patients with MG were compared to healthy controls (18). Of note, Tregs have functional plasticity. Huang et al. demonstrated that CD4+CD25-T-cells from patients with MG transformed into CD4+CD25+Tregs expressing FoxP3 when stimulated by IFN-γ in a dose-dependent fashion (19). Notably, these studies identified Tregs based on high CD25 expression, whereas the expression level of CD25 that identifies Treg was not standardized in the literature, and CD25 was also expressed in other T cells including effector T cells or T cells with pro-inflammatory properties (45). Accordingly, analyzing the number of Tregs merely according to CD25 expression may contradict results. Although there has been no agreement on whether the number of Tregs is altered yet, most findings reported functional defects in Tregs. For instance, Thiruppathi et al. demonstrated that the inhibitory function of responder T (Tresp) cells mediated by Tregs was disrupted in patients with MG, which correlated with reduced expression of FoxP3 (18). Balandina et al. found a severe functional defect in the regulatory activity of Tregs along with a decreased expression of FoxP3 (20). Therefore, the functional impairment of Tregs, both in the periphery and thymus, is more likely to be related to MG. FoxP3 has been shown to be a key player in the development and functional activity of Tregs (46). Thus, MG is associated with a decreased expression of FoxP3 on Tregs, which is corroborated by several studies (18, 20, 44, 47).

Briefly, abnormalities in the function or expression levels of Treg and Th17 cells exist in patients with MG. Therefore, an immune imbalance between Th17 and Tregs is a significant driver of MG. Based on the above-mentioned studies, improving this imbalance is a potential new avenue for treating MG. IL-6 plays pivotal roles in the regulation of the balance of Treg/Th17 cells (48). TGF-β helps induce the development of Treg and Th17 cells and solely drives the formation of Tregs from naive CD4+ T cells (49), whereas TGF-β together with IL-6 initiates Th17 lineage development (50). Anti-IL-6 monoclonal antibody has been reported to show good efficacy in patients with EAMG. Additionally, other studies have shown that rapamycin and artemisinin lessen motor symptoms in EAMG rats, with an ability of correcting Treg/Th17 imbalances (51, 52). Rapamycin is the most common inhibitor targeting mTOR, which inhibits the differentiation of Th17 cells (53) and induces the proliferation of Treg cells (54) by inhibiting the mTOR signaling pathway. Upon treatment with artemisinins, a decreased level of tumor necrosis factor-α (TNF-α) and IL-17+ cells in mononuclear cells (MNCs), and an increased level of transforming growth factor-β1 (TGF-β1) and Treg cells in MNCs were observed (52). These findings indicate that Rapamycin and artemisinin may be new choices for MG treatment, although investigations are still in its infancy. However, the reasons behind imbalances in Treg/Th17 cells in patients with MG are not well established. Conventionally, numerous cytokines are responsible for the maintenance of Treg/Th17 balance, and the abnormal expression of these cytokines due to thymoma or hyperplasia is one of the causes. Furthermore, gut microbiota is another critical regulator of Th17/Treg cell balance.

## Dysbiosis of Gut Microbiota and Microbial Metabolites in Patients With MG

The gut microbiota is defined as the microbial community colonizing the gut, including dominant bacteria, archaea, protists, fungi, and viruses. More than 90% of the bacteria in the adult gut belong to the phyla of Firmicutes and Bacteroidetes,while the rest belong to the phylum other phyla, such as Proteobacteria,Actinobacteria and Verrucomicrobia (55).

Gut microbiota colonizes the host and exerts an essential impact on host immune tolerance and immunostasis (56–59). Studies on germ-free (GF) mice and antibiotic-treated models have shown that gut microbiota can regulate the differentiation and development of immune cells (60). With advancements in corresponding research, it was found that gut microbiota participate in Th17 and Treg cell balance, and the potential mechanisms were also investigated (25). Specific components of the gut microbiome are involved in the production of pro-inflammatory cytokines and subsequent production of Th17 cells. Similarly, commensal bacteria and their metabolites can also promote the production of Tregs to facilitate immunosuppression. Under normal conditions, microbiome-mediated inflammatory factors and anti-inflammatory factors are in immune equilibrium. However, once gut dysbiosis occurs, host immune dysregulation may start, triggering chronic inflammatory disorders, such as inflammatory bowel disease (28), rheumatoid arthritis (27), multiple sclerosis (29), and MG (30). However, different diseases have different patterns of microbial dysbiosis.

Substantial evidence indicates that MG is accompanied by dysbiosis of gut microbiota and altered metabolites. Qiu et al. (61) demonstrated for the first time the decreased microbial diversity and altered community structures in MG *via* 16S rRNA sequencing technology, offering new insights on MG-relevant host-microbes interactions. Specifically, decreased phylum Firmicutes along with increased phylum Proteobacteria and Bacteroidetes were observed in the fecal samples of patients with MG. Accordingly, the ratio of Firmicutes/Bacteroidetes (F/B ratio) in patients with MG was markedly lower, which was consistent with the decreased F/B ratio observed in systemic lupus erythematosus and inflammatory bowel disease (62, 63). In fact, the F/B ratio can reflect a pro-inflammatory environment, where the inflammatory microbiota could damage the intestinal epithelium and subsequently trigger an immune response, leading to immunological imbalance. Regarding relative abundance at the genus level, Eubacterium and Clostridium were significantly lower in the MG group than in the control group, whereas Parasutterella and Streptococcus levels were significantly higher. Among these bacteria, Clostridium was the most depleted and comprised only approximately one-third in the healthy controls as analyzed by qPCR analyses. There is ample evidence that composition changes in the gut microbiota, particularly the abundance of Clostridium strains, have profound effects on the differentiation and development of T cells. Particularly, Clostridia can increase the expression of 2,3-dioxygenase, in turn, promotes the conversion of naive CD4+ T cells to Tregs by catalyzing the tryptophan-kynurenine (Trp-Kyn) metabolic pathway (64–66). Conventionally, Tregs have a profound effect on inhibiting autoreactive B cells and the production of anti-AChR autoantibodies (67). Therefore, we can speculate that if depleted, Clostridia can be restored, the number of Tregs will increase, consequently reducing autoimmune inflammatory responses in patients with MG and providing new insights on the treatment of MG at the microbial level. Moreover, it was also observed that *F. prausnitzii* was significantly reduced and that streptococcus was dramatically increased in the MG group at the genus level compared with the control groups; the control group would have the ability of activating peroxisome proliferator-activated receptor (PPARγ) through the suppression of immune cell function and certain signaling pathways, resulting in a tight balance of immune system responses (68, 69).

In another study (70), patients with MG were observed to harbor significantly higher proportions of Pasteurellaceae, Desulfovibrionaceae and Acidaminococcaceae compared to HCs. Moreover, patients with MG displayed a markedly lower relative abundance of the families of Bifdobacteriaceae and Verrucomicrobiaceae, as well as Leuconostocacceae, Flavobacteriaceae and Coriobacteriaceae. Results have shown an increased abundance of Bacteroidetes, among others, in patients with MG. A subsequent study in China found that the relative abundances of Bacteroidetes and Fusobacteria were increased in patients with MG, whereas those of Actinobacteria were decreased (71). A study aimed to identify the distinctive gut microbes in different subtypes of MG showed that patients with GMG harbored lower community richness and diversity and more severe gut microbial disturbances than patients with OMG. Compared with HCs, the families of Bacteroidaceae and Veillonellaceae were significantly more abundant, whereas families of Lachnospiraceae, Erysipelotrichaceae, Ruminococcaceae, Peptostreptococcaceae, Coriobacteriaceae, and Clostridiaceae_1 were scarcer in patients with MG (72). According to published literature, previous studies have suggested that Veillonellaceae and Bacteroidales are positively correlated with certain autoimmune disorders, whereas Ruminococcaceae and Lachnospiraceae, the common short chain fatty acids (SCFAs) producers (73), are negatively correlated with these diseases (74, 75). Recent research findings on the abnormal composition of gut microbiota in patients with MG are summarized in **Table 1**.

**Table 1:** Altered gut microbiota compositions in patients with MG.

Altered microbiota	References
Bacteroidetes↑, Actinobacteria↑, Verrucomicrobia↑,Desulfovibrionaceae↑, Acidaminococcaceae↑, Pasteurellaceae↑, Bifdobacteriaceae↓, Verrucomicrobiaceae↓, Coriobacteriaceae↓, Leuconostocacceae↓, and Flavobacteriaceae↓	Moris et al. (70)
Proteobacteria↑, Actinobacteria↑, Bacteroidetes↑, and Firmicutes↓	Qiu et al. (61)
Streptococcus↑, Parasutterella↑, Escherichia↑, Clostridium↓, Eubacterium↓, and Lactobacillus↓	
Firmicutes↑,Bacteroidetes↑, and Actinobacteria↓	Zheng et al. (71)
Bacteroidaceae↑,Veillonellaceae↑, and Prevotellaceae↑,	
Lachnospiraceae↓,Ruminococcaceae↓,Erysipelotrichaceae↓,Peptostreptococcaceae↓, and Clostridiaceae↓	
Bacteroidaceae↑, and Veillonellaceae↑	Tan et al. (72)
Lachnospiraceae↓,Erysipelotrichaceae↓,Ruminococcaceae↓,Peptostreptococcaceae↓,Coriobacteriaceae↓, and Clostridiaceae_1↓	

As one of beneficial microbial metabolites, short-chain fatty acids have been reported to have anti-inflammatory properties and be involved in the regulation of inflammatory responses (76–78). The total SCFAs content of patients with MG was reported to be lower than that of the HC cohort, with propionate and butyrate being significantly decreased (61). In another study, there was no difference in short chain fatty acids profiles between MG and controls (70). Taking into consideration the limited sample size and great inter-individual variability, this finding awaits further verification. Based on a previous finding proposed by Tan et al. that different subtypes of MG have different microbiota composition patterns (72), we can speculate that fecal metabolites were different among different subtypes. Overall, all the above mentioned studies demonstrated the presence of dysregulated gut microbiota and microbial metabolites in patients with MG, thus providing a new entry point into the pathogenesis of MG and helping us understand the full picture of myasthenia gravis to propose new therapeutic strategies.

To probe whether disturbed gut microbiota is involved in the occurrence and development of MG, fecal microbiota transplantation (FMT) was conducted by Peng et al. (71).

In experimental procedures, germ-free (GF) mice were implanted with the microbiota of patients with MG or healthy volunteers; subsequently these mice were immunized in a classical murine model of MG. Thus, MMb mice, HMb mice, and CMb mice were obtained, respectively. Compared to the mice colonized with HMb, the level of interferon gamma, tumor necrosis factor alpha, and IL-10 in the serum of MMb mice significantly increased. The MMb mice were observed to exhibit substantially impaired motor ability. Furthermore, these changes were reversed by colonization in the CMb mice. Taken together, these findings indicated that gut microbiota may probably be involved in the occurrence and development of MG. Moreover, experimental data have shown that the gut flora and its metabolites in MMb mice and patients with MG have roughly the same characteristics (71), suggesting that the gut microbiota may participate in the initiation and progress of MG by affecting host metabolism. This study provided novel knowledge on the pathogenesis of MG and a new scientific basis for the diagnosis and treatment of MG (79).

## Gut Microbiota Shapes the Th17/Treg Balance

The gut microbiome contributes importantly to the induction, maturation, and maintenance of the host immune system. Gut microbiota organisms could impact the balance of Th17/Treg cells, as reported by several studies on antibiotic-treated or germ-free animals (80–83). In contrast, metabolites produced by gut microbiota, particularly SCFAs have an effect on the induction of Tregs mediated by gut microbiota (79, 84, 85). However, certain gut microbes participate in the processes of the expansion and differentiation of immune cells (86, 87).

### Short-Chain Fatty Acids

SCFAs, a group of single-chain fatty acids with lengths less than 6 carbon chains, are produced by gut microbiota through the fermentation of dietary fiber, resistant starch, and other complex carbohydrates, as well as mucin (88, 89). These acids principally induce acetate, propionate, and butyrate, with an approximate molar ratio of 60:20:20 (90). The proportions of SCFAs can change depending on the diet, age, and diseases (91). There are differences in the generative process of the three most common SCFAs. Specifically, acetate is primarily produced by Bifidobacteria belonging to the Actinobacteria phylum during carbohydrate fermentation. Propionate is mainly generated by Bacteroides spp., and several Firmicutes bacteria *via* the succinate pathway (92). Butyrate is mostly produced by Clostridial clusters IV and XIVa *via* the pyruvate metabolism pathway (93).

In addition to providing an energy source for intestinal mucosal cells, SCFAs are important immune signaling molecules that influence the development of immune responses locally and distally. Multiple studies have demonstrated that SCFAs stimulate the differentiation and development of Tregs. Both acetate and propionate reduce the onset of allergic airway disease by upregulating the number of Tregs and enhancing Tregs function (94, 95). Furthermore, propionate is thought to be involved in the occurrence of allergic asthma by altering the gut microbiota and thus affecting the number and function of helper T (Th) cells (94). Extracellularly, SCFAs can serve as ligands for cell-surface G protein-coupled receptors (GPCRs) to indirectly regulate immune body function (96). Intracellularly, SCFAs regulate gene transcription by inhibiting histone deacetylases (HDACs) and enhancing histone acetyltransferase activity, thereby directly regulating immune function (97).

GPR43 is found on various cells, including macrophages, neutrophils, mast cells, epithelial cells and dendritic cells (DCs), in the intestine, especially in the colon (98–100). GPR43 on colonic T-cells have an impact on the induction and enhancement of the inhibitory effect of Tregs through epigenetic modifications (101). Propionate can reportedly increase the number of colonic Tregs, an effect dependent on GPR43 (101). GPR109A is present on macrophages and dendritic cells in addition to the intestinal epithelium and acts as a receptor for both butyrate and niacin. Niacin is not present at concentrations high enough to activate the receptor under physiologic conditions (102, 103). In the colonic lumen, a high concentration of butyrate produced by the microbiota acts as an endogenous agonist of GPR109A (104). In a Niacr1−/− (the gene encoding for GPR109A) mice study, it was observed that dendritic cells and colonic macrophages from mice suppressed the differentiation and proliferation of Tregs (105). GPR41 is widely expressed in the intestine, adipose tissue, and peripheral nervous system. SCFAs may also regulate the inflammatory response to maintain organismal health through the activation of GPR41. SCFAs inhibit dendritic cell proliferation and ameliorated allergic respiratory inflammation in wild-type mice, which did not occur in GPR41 knock-out mice (106). Briefly, the GPR signaling pathway is involved in the progression of many immune-related diseases, and SCFAs can play an important role in the regulation of immune response in the whole body through the activation of the GPR signal.

HDACs are a class of enzymes that remove acetyl groups from chromatin, thereby inhibiting transcription (107) and regulating a wide range of cellular functions, such as migration, metastasis (108, 109), and survival (110, 111). SCFAs can effectively suppress histone deacetylases (HDACs). Furthermore, the suppressive effect of SCFAs against HDACs occurs in a concentration-dependent manner (112). As early as 1979, Cousens et al. revealed that high concentrations of butyrate in rat hepatoma cells inhibited histone deacetylation by directly inhibiting HDAC activity (113). In addition to butyrate, propionate inhibited HDAC6 and HDAC9 expression in Tregs and enhanced histone acetylation in mice (101). Among all SCFAs, butyrate demonstrated the strongest inhibitory effects on HDACs. FoxP3 acts as a characteristic marker molecule that maintains the anti-inflammatory effects of Tregs, and HDAC9 can degrade it by affecting the degree of deacetylation of FoxP3 (114). SCFAs can promote the differentiation of Tregs by suppressing HDACs and enhancing their suppressive function. The mechanism may be that SCFAs suppress histone H3 lysine 27 acetylation (H3K27ac) in the CNS1 and CNS3 enhancers of the FoxP3 gene, thus promoting the expression of FoxP3, a process that may require the involvement of GPR43 (79, 101). In conclusion, SCFAs can regulate the differentiation and function of Tregs and modulate body immune tolerance by inhibiting HDACs.

### Commensal Gut Bacteria

Bacteroides fragilis, an anaerobic gram-negative gut microbe, activates the TLR signaling pathway to establish a host-microbe symbiotic relationship and influences the development and differentiation of T cells (115, 116). *B. fragilis* exerts its anti-inflammatory effects by acting on Tregs (117). Polysaccharide A (PSA) generated by *B. fragilis*, a microbe associated with molecular patterns, has been recognized by toll-like receptor 2 (TLR2) on Tregs to induce FoxP3+ Tregs and limit Th17 response, maintaining intestinal immune tolerance (118). Moreover, *B. fragilis*-derived PSA is recognized by dendritic cells (DCs) in the intestine, which produce IL-10, consequently promoting the production of Tregs (119).

Clostridia, a gram-positive spore-forming gut microbe in the small intestine, inhibits the release of IL-17 through the production of SCFAs, thereby reducing the differentiation to Th1 and Th17 cells and further expressing FoxP3, which exerts a Tregs-like induction effect (120). Clostridia colonizes the mucus layer near intestinal epithelial cells (IECs) (81) and increases the expression of 2,3-dioxygenase and matrix metalloproteinases (MMPs) in the epithelium (81). MMPs are known to be involved in the conversion of TGF-β from the latent to the active form, which promotes the differentiation of initial CD4+ T cells to antigen-specific colonic Tregs (121). Atarashi et al. used chloroform to treat the feces of conventionally reared mice to obtain 46 strains of Clostridium and colonized them in GF mice, inducing a robust accumulation of Tregs in the colons of GF mice (81). Subsequently, Atarashi et al. colonized 17 strains of Clostridia isolated from the human intrinsic microbiota into GF mice and found that they also enhanced Treg abundance. The oral administration of the combination of 17 strains was observed to attenuate allergic diarrhea and colitis in animal models (122).

**Figure 1:**
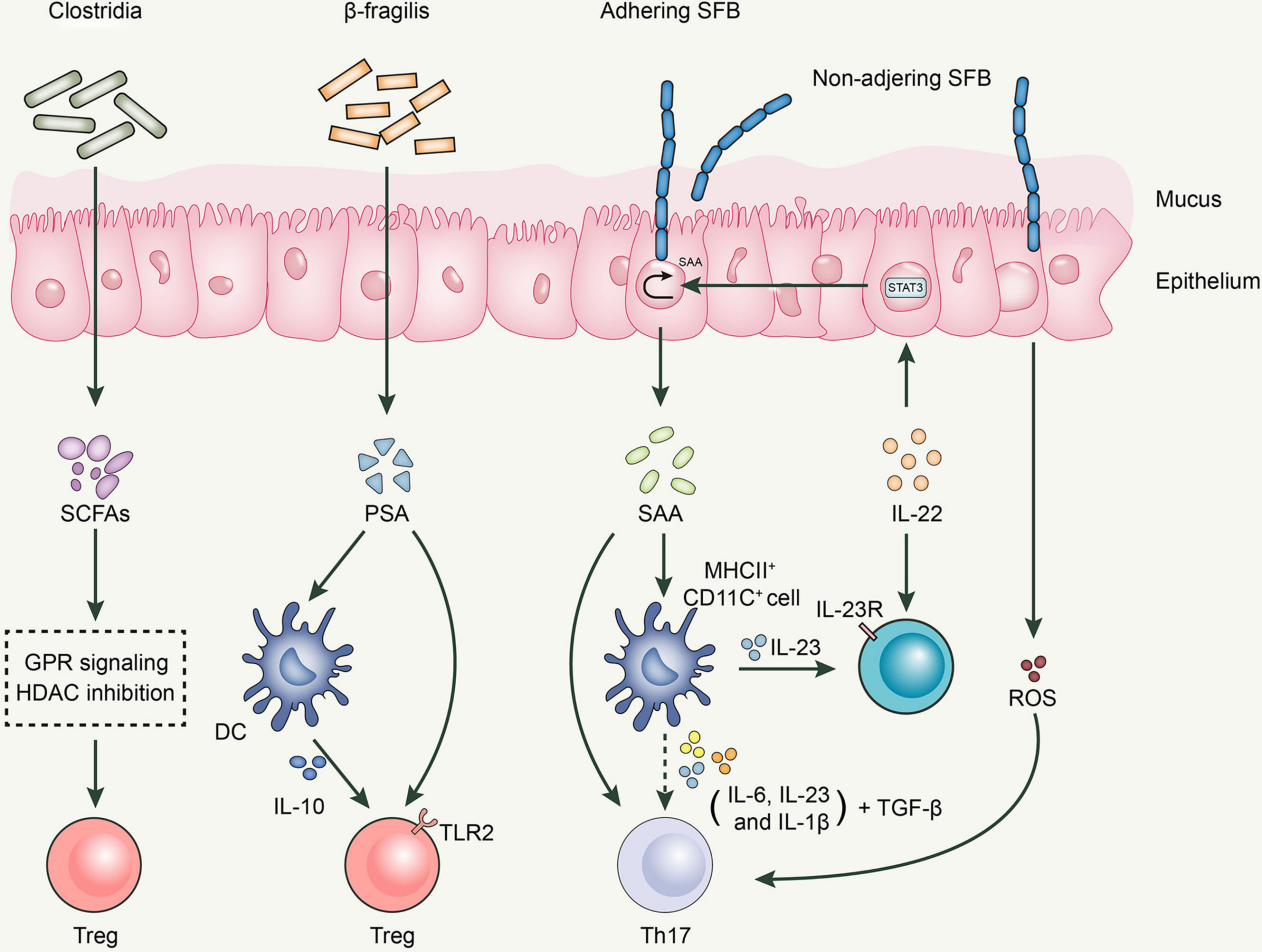
Roles of gut microbiota in Th17/Treg balance.

Segmented filamentous bacteria (SFB) is an anaerobic gram-positive spore-forming bacterium (123). SFB is essential for the proliferation and activation of Th17 cells, a process that cannot be separated from the adhesion of SFB to intestinal epithelial cells (124). The direct contact between SFB and intestinal epithelial cells induces the production of serum amyloid A proteins (SAA) and reactive oxygen species, creating an intestinal environment conducive to Th17 cell differentiation (124). SAA induces IL-23 production in DCs; subsequently, IL-23 triggers IL-22 secretion by type 3 innate lymphoid cells. IL-22 activates Stat3, which subsequently increases SAA expression (124, 125). Moreover, various cytokines secreted by CD11c+ cells in response to SAA stimulation, including IL-1β, IL-6, and IL-23, can boost the differentiation of Th17 cells together with TGF-β and SAA, with the latter being a vector of high-density lipoprotein (HDL) and retinol; therefore, it is speculated that SAA can deliver these immunomodulatory molecules to antigen-presenting cells (APCs) and T cells to regulate the body’s immune response. Thus, the SFB-mediated differentiation of Th17 cells is achieved through a complex network of interactions among multiple cells and cytokines (**Figure 1**).

Desulfovibrio is the major sulfate reducing bacteria (SRB) in the human gut (126). It not only inhibits the production and oxidation of butyrate, but also consumes the existing butyrate. Reduced butyrate level affects intestinal epithelial barrier function and promotes the expression of multiple inflammatory components, such as pathogen-associated molecular pattern (PAMP) or microbe-associated molecular pattern (MAMP) factors, jeopardizing the immune system of the body. Therefore, it is reasonable to presume that increased levels of desulfovibrio may contribute to the development of inflammation. When Figliuolo VR et al. examined the role of SRB in the gut immune response in GF mice, and in experimental colitis, they concluded that SRBs were capable of enhancing Th17 response in the mucosal immune system and promoting the occurrence and development of colitis (127).

## Potential Therapeutic Possibilities

Above all, the occurrence and development of MG is closely related to Th17/Treg cell-mediated immune imbalance. Perturbed microbial ecology is associated with the pathological process of MG, and regulating gut microbiota may improve the Th17/Treg cell imbalance. Therefore, microbiota-targeted interventions may potentially be effective in treating MG. Currently, probiotics, FMT, and other therapies targeting the gut microbiota have developed rapidly and have been widely used in clinical practice. According to relevant researches, novel therapeutic options for MG can be explored from the perspective of adjusting intestinal flora, improving pathological immune response and correcting immunity disorders.

### Probiotics

According to the Food and Agriculture Organization of the United Nations and the World Health Organization, probiotics are defined as “live microorganisms which, when administered in adequate amounts, confer a health benefit to the host (128)”. Probiotics are known to rebalance microbiota and modulate immune function through multiple mechanisms, including the direct inhibition of enteric pathogen activity, secretion of bacterial proteins by lowering luminal pH, induction of the defense mechanisms of the epithelium,and modification of immune regulation by reducing pro-inflammatory molecules and increasing anti-inflammatory factors (129).

Numerous animal and preclinical studies have demonstrated the potential benefits of probiotics in the therapy of diverse diseases, such as gastrointestinal (130) and neurological disorders (131). Bacterial species that are currently of research value as probiotics mainly belong to the genus Lactobacillus and Bifidobacterium. For example, Park et al. demonstrated the low levels of proinflammatory cytokines produced by Th17 cells in mouse experimental colitis models following the oral administration of Lactobacillus acidophilus. *In-vitro*, *L. acidophilus* induced Tregs and promoted the production of IL-10, whereas IL-17 secretion was inhibited in splenocytes (130). These results suggested that *L. acidophilus* could treat inflammatory bowel disease by regulating the Th17/Treg balance. A study has shown that the oral administration of IRT5 (a mixture of five probiotics) to experimental autoimmune encephalomyelitis mice not only enhanced Treg abundance, but reduced the number of Th17 cells in superficial lymph nodes. IRT5 dramatically suppressed experimental autoimmune encephalomyelitis when used as a pretreatment before disease onset. Moreover, probiotic treatment delayed disease progression (131). Following 8 weeks of orally administering mixed strains of bifidobacteria and lactic acid bacteria, atopic skin irritation was reduced in atopic dermatitis mouse models (132). Modulating gut microbiota by the ingestion of probiotics has been demonstrated to impact the development of mucosal and systemic immune responses, thereby altering immune profiles and immune homeostasis and positively influencing autoimmune diseases. In recent years, many international studies have demonstrated the use of probiotics to regulate MG. It was observed that the prophylactic administration of IRT5 modulated the disease course and reduced the titer of anti-AChR antibody and the level of certain pro‐inflammatory factors in EAMG rats (133). A study with applications of two bifidobacteria or lactobacilli strains in a Lewis rat model also resulted in promising results (30). A subsequent study (134) reported the clinical efficacy of bifidobacterium on the course of EAMG and found that the immunomodulatory properties of viable bifidobacteria relied on the maintenance of the integrity of bacterial proteins and DNA sequences, which is denatured when exposed to high temperatures (135). Together, these studies support the effectiveness of probiotics in different immune-mediated inflammatory conditions and their possible use as a treatment in patients with MG to modulate the immune system and contribute to the restoration of the immunological tolerance to AChR. Taken together, these research studies support the validity of probiotics in different autoimmune and inflammatory disease and their use as an attractive therapeutic intervention for MG to regulate the immune microenvironment.

However, the number of studies on the application of probiotics in MG is currently insufficient, resulting in an incomplete understanding of the mechanisms explaining how probiotics improve immune disorders in myasthenia gravis. In the future, more animal and clinical studies are needed to provide detailed information and reliable evidence on the beneficial effects of probiotics.

### Fecal Microbiota Transplantation

FMT refers to the administration of intestinal microbes from a healthy donor into a recipient to reconstruct the intestinal microecology of the recipient (136). If the gut microbiota plays a causal role in disease pathophysiology, altering the microbiota may influence disease course. However, in most cases, a single microorganism is not likely to be a causal pathogen or missing beneficial microbe. Therefore, an advantage of FMT over probiotics is the introduction of a complete healthy gut microbiota. The successful application of FMT in recurrent or refractory Clostridioides difficile infection has gradually became a research hotspot on biomedicine and clinical medicine.

With thorough research, the gut microbiota has been found to play a significant role in the pathophysiology of neurological disorders, and FMT is likely be a promising treatment option. In the field of neurological disease, the positive effect of FMT is supported by several animal studies or described in some case reports. Using the Parkinson’s disease (PD) model, Sun et al. demonstrated that FMT reduced gut microbial dysbiosis, decreased fecal SCFAs, and alleviated physical impairment (137). Sun et al. showed that FMT treatment improved cognitive deficits in APPswe/PS1dE9 transgenic mice, which was attributed to the reduction of the brain deposition of amyloid-β (138). There are few studies on the use of FMT in patients with MG. In a study aimed at investigating whether the microbiota participated in MG pathogenesis, the impaired locomotion ability of MMb mice was restored by cocolonizing with HMb, indicating a bright future for such applications in patients with MG (71).

Currently, studies on the application of FMT have been conducted only in animals. However, the immunoreactivity observed in animal models are quite different from that in humans. Thus, a large double-blinded randomized controlled human trial should be performed.

## Conclusions and Perspectives

As the most prevalent neuromuscular junction autoimmune disease, MG is characterized by increased numbers of Th17 cells and Treg dysfunctionalities. Regulating the cytokines, receptors, and signal transduction pathways involved in the Th17/Treg immunobalance axis will provide a viable strategy for the treatment of MG. Recently, scientists have gradually realized the role of gut microbiota in affecting the balance of Th17/Treg cells. Accordingly, correcting Th17/Treg imbalances by regulating the composition of the gut microbiota would be beneficial for MG treatment.

Probiotics are a class of active microorganisms that are beneficial to the host, with a favorable effect on the microbial ecological balance of the host. Probiotics contribute to modulating immune responses and fostering immunological surveillance. Certain probiotic strains reportedly promote the production of Tregs. Therefore, probiotics may be an attractive option to treat myasthenia gravis characterized by a deficiency of Treg. It is important to be alert to the fact that certain strains of probiotics may exacerbate the disease; therefore, it is critical to fully characterize the immunological profile and dosage of probiotics to exert their beneficial effects.

FMT has an advantage over probiotics due to the introduction of a complete healthy gut microbiota. Due to the successful application of FMT in different areas of biomedicine, it is also currently being investigated in the domain of neurological disease with dysbiosis of gut microbiota.

Considering the low cost and high efficiency of FMT, the application of FMT for diseases with Th17/Treg cells imbalances is practical at the theoretical level. However, this therapeutic approach is unpredictable, and a single species microorganism transfer may be more effective; however, the detailed elucidation of the mechanisms involved is needed.

## Author Contributions

PC and XT conceived the review. PC wrote the manuscript. XT revised the manuscript. All authors contributed to the article and approved the submitted version.

## Conflict of Interest

The authors declare that the research was conducted in the absence of any commercial or financial relationships that could be construed as a potential conflict of interest.

## Publisher’s Note

All claims expressed in this article are solely those of the authors and do not necessarily represent those of their affiliated organizations, or those of the publisher, the editors and the reviewers. Any product that may be evaluated in this article, or claim that may be made by its manufacturer, is not guaranteed or endorsed by the publisher.

## References

[1] Gilhus NE Tzartos S Evoli A Palace J Burns TM Verschuuren J. Myasthenia Gravis. Nat Rev Dis Primers (2019) 5(1):30. doi: 10.1038/s41572-019-0079-y 31048702

[2] Dalakas MC. Immunotherapy in Myasthenia Gravis in the Era of Biologics. Nat Rev Neurol (2019) 15(2):113–24. doi: 10.1038/s41582-018-0110-z 30573759

[3] Gilhus NE Skeie GO Romi F Lazaridis K Zisimopoulou P Tzartos S. Myasthenia Gravis - Autoantibody Characteristics and Their Implications for Therapy. Nat Rev Neurol (2016) 12(5):259–68. doi: 10.1038/nrneurol.2016.44 27103470

[4] Gilhus NE Verschuuren JJ. Myasthenia Gravis: Subgroup Classification and Therapeutic Strategies. Lancet Neurol (2015) 14(10):1023–36. doi: 10.1016/s1474-4422(15)00145-3 26376969

[5] Carr AS Cardwell CR McCarron PO McConville J. A Systematic Review of Population Based Epidemiological Studies in Myasthenia Gravis. BMC Neurol (2010) 10:46. doi: 10.1186/1471-2377-10-46 20565885 PMC2905354

[6] Ramanujam R Pirskanen R Ramanujam S Hammarström L. Utilizing Twins Concordance Rates to Infer the Predisposition to Myasthenia Gravis. Twin Res Hum Genet (2011) 14(2):129–36. doi: 10.1375/twin.14.2.129 21425894

[7] Li F Yuan W Wu X. Association of CTLA-4 Polymorphisms With Increased Risks of Myasthenia Gravis. Ann Hum Genet (2018) 82(6):358–69. doi: 10.1111/ahg.12262 30009380

[8] Ströbel P Moritz R Leite MI Willcox N Chuang WY Gold R. The Ageing and Myasthenic Thymus: A Morphometric Study Validating a Standard Procedure in the Histological Workup of Thymic Specimens. J Neuroimmunol (2008) 201:64–73. doi: 10.1016/j.jneuroim.2008.06.017 18657325

[9] Le Panse R Cizeron-Clairac G Cuvelier M Truffault F Bismuth J Nancy P. Regulatory and Pathogenic Mechanisms in Human Autoimmune Myasthenia Gravis. Ann N Y Acad Sci (2008) 1132:135–42. doi: 10.1196/annals.1405.019 18567863

[10] Zhang Y Zhang Y Gu W He L Sun B. Th1/Th2 Cell’s Function in Immune System. Adv Exp Med Biol (2014) 841:45–65. doi: 10.1007/978-94-017-9487-9_3 25261204

[11] Yi JS Guptill JT Stathopoulos P Nowak RJ O’Connor KC. B Cells in the Pathophysiology of Myasthenia Gravis. Muscle Nerve (2018) 57(2):172–84. doi: 10.1002/mus.25973 PMC5767142 28940642

[12] Scott DW. Driving CARs to BARs: The Winding Road to Specific Regulatory T Cells for Tolerance. Front Immunol (2021) 12:742719. doi: 10.3389/fimmu.2021.742719 34552599 PMC8450509

[13] Diller ML Kudchadkar RR Delman KA Lawson DH Ford ML. Balancing Inflammation: The Link Between Th17 and Regulatory T Cells. Mediators Inflamm (2016) 2016:6309219. doi: 10.1155/2016/6309219 27413254 PMC4930807

[14] Schaffert H Pelz A Saxena A Losen M Meisel A Thiel A. IL-17-Producing CD4(+) T Cells Contribute to the Loss of B-Cell Tolerance in Experimental Autoimmune Myasthenia Gravis. Eur J Immunol (2015) 45(5):1339–47. doi: 10.1002/eji.201445064 25676041

[15] Aguilo-Seara G Xie Y Sheehan J Kusner LL Kaminski HJ. Ablation of IL-17 Expression Moderates Experimental Autoimmune Myasthenia Gravis Disease Severity. Cytokine (2017) 96:279–85. doi: 10.1016/j.cyto.2017.05.008 28599246

[16] Roche JC Capablo JL Larrad L Gervas-Arruga J Ara JR Sánchez A. Increased Serum Interleukin-17 Levels in Patients With Myasthenia Gravis. Muscle Nerve (2011) 44(2):278–80. doi: 10.1002/mus.22070 21755509

[17] Hu Y Wang J Rao J Xu X Cheng Y Yan L. Comparison of Peripheral Blood B Cell Subset Ratios and B Cell-Related Cytokine Levels Between Ocular and Generalized Myasthenia Gravis. Int Immunopharmacol (2020) 80:106130. doi: 10.1016/j.intimp.2019.106130 31978800

[18] Thiruppathi M Rowin J Ganesh B Sheng JR Prabhakar BS Meriggioli MN. Impaired Regulatory Function in Circulating CD4(+)CD25(high)CD127(low/-) T Cells in Patients With Myasthenia Gravis. Clin Immunol (2012) 145(3):209–23. doi: 10.1016/j.clim.2012.09.012 PMC3501560 23110942

[19] Huang S Wang W Chi L. Feasibility of Up-Regulating CD4(+)CD25(+) Tregs by IFN-γ in Myasthenia Gravis Patients. BMC Neurol (2015) :15:163. doi: 10.1186/s12883-015-0419-9 26347149 PMC4562356

[20] Balandina A Lécart S Dartevelle P Saoudi A Berrih-Aknin S. Functional Defect of Regulatory CD4(+)CD25+ T Cells in the Thymus of Patients With Autoimmune Myasthenia Gravis. Blood (2005) 105(2):735–41. doi: 10.1182/blood-2003-11-3900 PMC1847365 15454488

[21] Wu HJ Wu E. The Role of Gut Microbiota in Immune Homeostasis and Autoimmunity. Gut Microbes (2012) 3(1):4–14. doi: 10.4161/gmic.19320 22356853 PMC3337124

[22] Belkaid Y Hand TW. Role of the Microbiota in Immunity and Inflammation. Cell (2014) 157(1):121–41. doi: 10.1016/j.cell.2014.03.011 PMC4056765 24679531

[23] Kim D Yoo SA Kim WU. Gut Microbiota in Autoimmunity: Potential for Clinical Applications. Arch Pharm Res (2016) 39(11):1565–76. doi: 10.1007/s12272-016-0796-7 27444041

[24] Gopalakrishnan V Helmink BA Spencer CN Reuben A Wargo JA. The Influence of the Gut Microbiome on Cancer, Immunity, and Cancer Immunotherapy. Cancer Cell (2018) 33(4):570–80. doi: 10.1016/j.ccell.2018.03.015 PMC6529202 29634945

[25] Omenetti S Pizarro TT. The Treg/Th17 Axis: A Dynamic Balance Regulated by the Gut Microbiome. Front Immunol (2015) 6:639. doi: 10.3389/fimmu.2015.00639 26734006 PMC4681807

[26] Frati F Salvatori C Incorvaia C Bellucci A Di Cara G Marcucci F. The Role of the Microbiome in Asthma: The Gut⁻Lung Axis. Int J Mol Sci (2018) 20(1):123. doi: 10.3390/ijms20010123 PMC6337651 30598019

[27] Horta-Baas G Romero-Figueroa MDS Montiel-Jarquín AJ Pizano-Zárate ML García-Mena J Ramírez-Durán N. Intestinal Dysbiosis and Rheumatoid Arthritis: A Link Between Gut Microbiota and the Pathogenesis of Rheumatoid Arthritis. J Immunol Res (2017) 2017:4835189. doi: 10.1155/2017/4835189 28948174 PMC5602494

[28] Nishida A Inoue R Inatomi O Bamba S Naito Y Andoh A. Gut Microbiota in the Pathogenesis of Inflammatory Bowel Disease. Clin J Gastroenterol (2018) 11(1):1–10. doi: 10.1007/s12328-017-0813-5 29285689

[29] Shahi SK Freedman SN Mangalam AK. Gut Microbiome in Multiple Sclerosis: The Players Involved and the Roles They Play. Gut Microbes (2017) 8(6):607–15. doi: 10.1080/19490976.2017.1349041 PMC5730390 28696139

[30] Rinaldi E Consonni A Guidesi E Elli M Mantegazza R Baggi F. Gut Microbiota and Probiotics: Novel Immune System Modulators in Myasthenia Gravis? Ann N Y Acad Sci (2018) 1413(1):49–58. doi: 10.1111/nyas.13567 29341125

[31] Beam A Clinger E Hao L. Effect of Diet and Dietary Components on the Composition of the Gut Microbiota. Nutrients (2021) 13(8):2795. doi: 10.3390/nu13082795 34444955 PMC8398149

[32] Yang J Wei H Zhou Y Szeto CH Li C Lin Y. High-Fat Diet Promotes Colorectal Tumorigenesis Through Modulating Gut Microbiota and Metabolites. Gastroenterology (2021) 162(1):135–49.e2. doi: 10.1053/j.gastro.2021.08.041 34461052

[33] Guilleminault L Williams EJ Scott HA Berthon BS Jensen M Wood LG. Diet and Asthma: Is It Time to Adapt Our Message? Nutrients (2017) 9(11):1227. doi: 10.3390/nu9111227 PMC5707699 29117118

[34] Frontela-Saseta C González-Bermúdez CA García-Marcos L. Diet: A Specific Part of the Western Lifestyle Pack in the Asthma Epidemic. J Clin Med (2020) 9(7):2063. doi: 10.3390/jcm9072063 PMC7408793 32630168

[35] Nalbant A Eskier D. Genes Associated With T Helper 17 Cell Differentiation and Function. Front Biosci (Elite Ed) (2016) 8:427–35. doi: 10.2741/e777 27100349

[36] Zhu J Paul WE. CD4 T Cells: Fates, Functions, and Faults. Blood (2008) 112(5):1557–69. doi: 10.1182/blood-2008-05-078154 PMC2518872 18725574

[37] Korn T Bettelli E Oukka M Kuchroo VK. IL-17 and Th17 Cells. Annu Rev Immunol (2009) 27:485–517. doi: 10.1146/annurev.immunol.021908.132710 19132915

[38] Fossiez F Banchereau J Murray R Van Kooten C Garrone P Lebecque S. Interleukin-17. Int Rev Immunol (1998) 16(5-6):541–51. doi: 10.3109/08830189809043008 9646176

[39] Jovanovic DV Di Battista JA Martel-Pelletier J Jolicoeur FC He Y Zhang M. IL-17 Stimulates the Production and Expression of Proinflammatory Cytokines, IL-Beta and TNF-Alpha, by Human Macrophages. J Immunol (1998) 160(7):3513–21. 9531313

[40] Masuda M Matsumoto M Tanaka S Nakajima K Yamada N Ido N. Clinical Implication of Peripheral CD4+CD25+ Regulatory T Cells and Th17 Cells in Myasthenia Gravis Patients. J Neuroimmunol (2010) 225(1-2):123–31. doi: 10.1016/j.jneuroim.2010.03.016 20472307

[41] Rodríguez-Perea AL Arcia ED Rueda CM Velilla PA. Phenotypical Characterization of Regulatory T Cells in Humans and Rodents. Clin Exp Immunol (2016) 185(3):281–91. doi: 10.1111/cei.12804 PMC4991523 27124481

[42] Tsuji-Takayama K Suzuki M Yamamoto M Harashima A Okochi A Otani T. The Production of IL-10 by Human Regulatory T Cells is Enhanced by IL-2 Through a STAT5-Responsive Intronic Enhancer in the IL-10 Locus. J Immunol (2008) 181(6):3897–905. doi: 10.4049/jimmunol.181.6.3897 18768844

[43] Gavin MA Rasmussen JP Fontenot JD Vasta V Manganiello VC Beavo JA. Foxp3-Dependent Programme of Regulatory T-Cell Differentiation. Nature (2007) 445(7129):771–5. doi: 10.1038/nature05543 17220874

[44] Xu WH Zhang AM Ren MS Zhang XD Wang F Xu XC. Changes of Treg-Associated Molecules on CD4+CD25 +Treg Cells in Myasthenia Gravis and Effects of Immunosuppressants. J Clin Immunol (2012) 32(5):975–83. doi: 10.1007/s10875-012-9685-0 22467037

[45] Baecher-Allan C Brown JA Freeman GJ Hafler DA. CD4+CD25high Regulatory Cells in Human Peripheral Blood. J Immunol (2001) 167(3):1245–53. doi: 10.4049/jimmunol.167.3.1245 11466340

[46] Josefowicz SZ Lu LF Rudensky AY. Regulatory T Cells: Mechanisms of Differentiation and Function. Annu Rev Immunol (2012) 30:531–64. doi: 10.1146/annurev.immunol.25.022106.141623 PMC6066374 22224781

[47] Zhang J Chen Y Jia G Chen X Lu J Yang H. FOXP3 -3279 and IVS9+459 Polymorphisms are Associated With Genetic Susceptibility to Myasthenia Gravis. Neurosci Lett (2013) 534:274–8. doi: 10.1016/j.neulet.2012.11.048 23228687

[48] Kimura A Kishimoto T. IL-6: Regulator of Treg/Th17 Balance. Eur J Immunol (2010) 40(7):1830–5. doi: 10.1002/eji.201040391 20583029

[49] Chen W Jin W Hardegen N Lei KJ Li L Marinos N. Conversion of Peripheral CD4+CD25- Naive T Cells to CD4+CD25+ Regulatory T Cells by TGF-Beta Induction of Transcription Factor Foxp3. J Exp Med (2003) 198(12):1875–86. doi: 10.1084/jem.20030152 PMC2194145 14676299

[50] Bettelli E Carrier Y Gao W Korn T Strom TB Oukka M. Reciprocal Developmental Pathways for the Generation of Pathogenic Effector TH17 and Regulatory T Cells. Nature (2006) 441(7090):235–8. doi: 10.1038/nature04753 16648838

[51] Jing F Yang F Cui F Chen Z Ling L Huang X. Rapamycin Alleviates Inflammation and Muscle Weakness, While Altering the Treg/Th17 Balance in a Rat Model of Myasthenia Gravis. Biosci Rep (2017) 37(4):BSR20170767. doi: 10.1042/bsr20170767 28655853 PMC5518538

[52] Chen W Li FF Li C Sui JK Meng QF Li XL. Artemisinin Ameliorates the Symptoms of Experimental Autoimmune Myasthenia Gravis by Regulating the Balance of TH1 Cells, TH17 Cells and Treg Cells. J Biol Regul Homeost Agents (2018) 32(5):1217–23. 30334416

[53] Stepkowski SM. Preclinical Results of Sirolimus Treatment in Transplant Models. Transplant Proc (2003) 35(3 Suppl):219s–26s. doi: 10.1016/s0041-1345(03)00222-7 12742499

[54] Feuerer M Hill JA Mathis D Benoist C. Foxp3+ Regulatory T Cells: Differentiation, Specification, Subphenotypes. Nat Immunol (2009) 10(7):689–95. doi: 10.1038/ni.1760 19536194

[55] Human Microbiome Project Consortium. Structure, Function and Diversity of the Healthy Human Microbiome. Nature (2012) 486(7402):207–14. doi: 10.1038/nature11234 PMC3564958 22699609

[56] Pacaud M Colas L Brouard S. Microbiota and Immunoregulation: A Focus on Regulatory B Lymphocytes and Transplantation. Am J Transplant (2021) 21(7):2341–7. doi: 10.1111/ajt.16522 33559282

[57] Chi M Ma K Wang J Ding Z Li Y Zhu S. The Immunomodulatory Effect of the Gut Microbiota in Kidney Disease. J Immunol Res (2021) 2021:5516035. doi: 10.1155/2021/5516035 34095319 PMC8140847

[58] Riazi-Rad F Behrouzi A Mazaheri H Katebi A Ajdary S. Impact of Gut Microbiota on Immune System. Acta Microbiol Immunol Hung (2021) 68(3):135–44. doi: 10.1556/030.2021.01532 34375301

[59] La Flamme AC Milling S. Immunological Partners: The Gut Microbiome in Homeostasis and Disease. Immunology (2020) 161(1):1–3. doi: 10.1111/imm.13247 32851647 PMC7450166

[60] Lee N Kim WU. Microbiota in T-Cell Homeostasis and Inflammatory Diseases. Exp Mol Med (2017) 49(5):e340. doi: 10.1038/emm.2017.36 28546563 PMC5454441

[61] Qiu D Xia Z Jiao X Deng J Zhang L Li J. Altered Gut Microbiota in Myasthenia Gravis. Front Microbiol (2018) 9:2627. doi: 10.3389/fmicb.2018.02627 30483222 PMC6241162

[62] Stojanov S Berlec A Štrukelj B. The Influence of Probiotics on the Firmicutes/Bacteroidetes Ratio in the Treatment of Obesity and Inflammatory Bowel Disease. Microorganisms (2020) 8(11):1715. doi: 10.3390/microorganisms8111715 PMC7692443 33139627

[63] Gerges MA Esmaeel NE Makram WK Sharaf DM Gebriel MG. Altered Profile of Fecal Microbiota in Newly Diagnosed Systemic Lupus Erythematosus Egyptian Patients. Int J Microbiol (2021) 2021:9934533. doi: 10.1155/2021/9934533 34257666 PMC8249152

[64] Correale J. Immunosuppressive Amino-Acid Catabolizing Enzymes in Multiple Sclerosis. Front Immunol (2020) 11:600428. doi: 10.3389/fimmu.2020.600428 33552055 PMC7855700

[65] Cong Y Feng T Fujihashi K Schoeb TR Elson CO. A Dominant, Coordinated T Regulatory Cell-IgA Response to the Intestinal Microbiota. Proc Natl Acad Sci U S A (2009) 106(46):19256–61. doi: 10.1073/pnas.0812681106 PMC2780781 19889972

[66] Kuhn KA Stappenbeck TS. Peripheral Education of the Immune System by the Colonic Microbiota. Semin Immunol (2013) 25(5):364–9. doi: 10.1016/j.smim.2013.10.002 PMC3864652 24169518

[67] Shin DS Jordan A Basu S Thomas RM Bandyopadhyay S de Zoeten EF. Regulatory T Cells Suppress CD4+ T Cells Through NFAT-Dependent Transcriptional Mechanisms. EMBO Rep (2014) 15(9):991–9. doi: 10.15252/embr.201338233 PMC4198043 25074018

[68] Agafonoff S Hawke I Khadra M Munnings V Notaras L Wadhwa S. The Influence of Age and Gender on Normal Appendicectomy Rates. Aust N Z J Surg (1987) 57(11):843–6. doi: 10.1111/j.1445-2197.1987.tb01277.x 3439927

[69] Nettleford SK Prabhu KS. Selenium and Selenoproteins in Gut Inflammation-A Review. Antioxid (Basel) (2018) 7(3):36. doi: 10.3390/antiox7030036 PMC5874522 29494512

[70] Moris G Arboleya S Mancabelli L Milani C Ventura M de Los Reyes-Gavilán CG. Fecal Microbiota Profile in a Group of Myasthenia Gravis Patients. Sci Rep (2018) 8(1):14384. doi: 10.1038/s41598-018-32700-y 30258104 PMC6158187

[71] Zheng P Li Y Wu J Zhang H Huang Y Tan X. Perturbed Microbial Ecology in Myasthenia Gravis: Evidence From the Gut Microbiome and Fecal Metabolome. Adv Sci (Weinh) (2019) 6(18):1901441. doi: 10.1002/advs.201901441 31559142 PMC6755540

[72] Tan X Huang Y Chai T Zhao X Li Y Wu J. Differential Gut Microbiota and Fecal Metabolites Related With the Clinical Subtypes of Myasthenia Gravis. Front Microbiol (2020) 11:564579. doi: 10.3389/fmicb.2020.564579 33013794 PMC7506099

[73] Yilmaz B Juillerat P Øyås O Ramon C Bravo FD Franc Y. Microbial Network Disturbances in Relapsing Refractory Crohn’s Disease. Nat Med (2019) 25(2):323–36. doi: 10.1038/s41591-018-0308-z 30664783

[74] Gevers D Kugathasan S Denson LA Vázquez-Baeza Y Van Treuren W Ren B. The Treatment-Naive Microbiome in New-Onset Crohn’s Disease. Cell Host Microbe (2014) 15(3):382–92. doi: 10.1016/j.chom.2014.02.005 PMC4059512 24629344

[75] Clemente JC Manasson J Scher JU. The Role of the Gut Microbiome in Systemic Inflammatory Disease. Bmj (2018) 360:j5145. doi: 10.1136/bmj.j5145 29311119 PMC6889978

[76] Maeda T Towatari M Kosugi H Saito H. Up-Regulation of Costimulatory/Adhesion Molecules by Histone Deacetylase Inhibitors in Acute Myeloid Leukemia Cells. Blood (2000) 96(12):3847–56. doi: 10.1182/blood.V96.12.3847 11090069

[77] Lührs H Gerke T Müller JG Melcher R Schauber J Boxberge F. Butyrate Inhibits NF-kappaB Activation in Lamina Propria Macrophages of Patients With Ulcerative Colitis. Scand J Gastroenterol (2002) 37(4):458–66. doi: 10.1080/003655202317316105 11989838

[78] Miyake S Kim S Suda W Oshima K Nakamura M Matsuoka T. Dysbiosis in the Gut Microbiota of Patients With Multiple Sclerosis, With a Striking Depletion of Species Belonging to Clostridia XIVa and IV Clusters. PloS One (2015) 10(9):e0137429. doi: 10.1371/journal.pone.0137429 26367776 PMC4569432

[79] Furusawa Y Obata Y Fukuda S Endo TA Nakato G Takahashi D. Commensal Microbe-Derived Butyrate Induces the Differentiation of Colonic Regulatory T Cells. Nature (2013) 504(7480):446–50. doi: 10.1038/nature12721 24226770

[80] Atarashi K Nishimura J Shima T Umesaki Y Yamamoto M Onoue M. ATP Drives Lamina Propria T(H)17 Cell Differentiation. Nature (2008) 455(7214):808–12. doi: 10.1038/nature07240 18716618

[81] Atarashi K Tanoue T Shima T Imaoka A Kuwahara T Momose Y. Induction of Colonic Regulatory T Cells by Indigenous Clostridium Species. Science (2011) 331(6015):337–41. doi: 10.1126/science.1198469 PMC3969237 21205640

[82] Hall JA Bouladoux N Sun CM Wohlfert EA Blank RB Zhu Q. Commensal DNA Limits Regulatory T Cell Conversion and is a Natural Adjuvant of Intestinal Immune Responses. Immunity (2008) 29(4):637–49. doi: 10.1016/j.immuni.2008.08.009 PMC2712925 18835196

[83] Ivanov II Frutos Rde L Manel N Yoshinaga K Rifkin DB Sartor RB. Specific Microbiota Direct the Differentiation of IL-17-Producing T-Helper Cells in the Mucosa of the Small Intestine. Cell Host Microbe (2008) 4(4):337–49. doi: 10.1016/j.chom.2008.09.009 PMC2597589 18854238

[84] Arpaia N Campbell C Fan X Dikiy S van der Veeken J deRoos P. Metabolites Produced by Commensal Bacteria Promote Peripheral Regulatory T-Cell Generation. Nature (2013) 504(7480):451–5. doi: 10.1038/nature12726 PMC3869884 24226773

[85] Luu M Visekruna A. Short-Chain Fatty Acids: Bacterial Messengers Modulating the Immunometabolism of T Cells. Eur J Immunol (2019) 49(6):842–8. doi: 10.1002/eji.201848009 31054154

[86] Ray S De Salvo C Pizarro TT. Central Role of IL-17/Th17 Immune Responses and the Gut Microbiota in the Pathogenesis of Intestinal Fibrosis. Curr Opin Gastroenterol (2014) 30(6):531–8. doi: 10.1097/mog.0000000000000119 PMC4512208 25255234

[87] Zákostelská Z Málková J Klimešová K Rossmann P Hornová M Novosádová I. Intestinal Microbiota Promotes Psoriasis-Like Skin Inflammation by Enhancing Th17 Response. PloS One (2016) 11(7):e0159539. doi: 10.1371/journal.pone.0159539 27434104 PMC4951142

[88] Canfora EE Jocken JW Blaak EE. Short-Chain Fatty Acids in Control of Body Weight and Insulin Sensitivity. Nat Rev Endocrinol (2015) 11(10):577–91. doi: 10.1038/nrendo.2015.128 26260141

[89] Marchesi JR Adams DH Fava F Hermes GD Hirschfield GM Hold G. The Gut Microbiota and Host Health: A New Clinical Frontier. Gut (2016) 65(2):330–9. doi: 10.1136/gutjnl-2015-309990 PMC4752653 26338727

[90] Fernandes J Su W Rahat-Rozenbloom S Wolever TM Comelli EM. Adiposity, Gut Microbiota and Faecal Short Chain Fatty Acids are Linked in Adult Humans. Nutr Diabetes (2014) 30 4(6):e121. doi: 10.1038/nutd.2014.23 PMC4079931 24979150

[91] Ratajczak W Rył A Mizerski A Walczakiewicz K Sipak O Laszczyńska M. Immunomodulatory Potential of Gut Microbiome-Derived Short-Chain Fatty Acids (SCFAs). Acta Biochim Pol (2019) 66(1):1–12. doi: 10.18388/abp.2018_2648 30831575

[92] Reichardt N Duncan SH Young P Belenguer A McWilliam Leitch C Scott KP. Phylogenetic Distribution of Three Pathways for Propionate Production Within the Human Gut Microbiota. Isme J (2014) 8(6):1323–35. doi: 10.1038/ismej.2014.14 PMC4030238 24553467

[93] Pryde SE Duncan SH Hold GL Stewart CS Flint HJ. The Microbiology of Butyrate Formation in the Human Colon. FEMS Microbiol Lett (2002) 217(2):133–9. doi: 10.1111/j.1574-6968.2002.tb11467.x 12480096

[94] Trompette A Gollwitzer ES Yadava K Sichelstiel AK Sprenger N Ngom-Bru C. Gut Microbiota Metabolism of Dietary Fiber Influences Allergic Airway Disease and Hematopoiesis. Nat Med (2014) 20(2):159–66. doi: 10.1038/nm.3444 24390308

[95] Thorburn AN McKenzie CI Shen S Stanley D Macia L Mason LJ. Evidence That Asthma is a Developmental Origin Disease Influenced by Maternal Diet and Bacterial Metabolites. Nat Commun (2015) 6:7320. doi: 10.1038/ncomms8320 26102221

[96] Tan J McKenzie C Potamitis M Thorburn AN Mackay CR Macia L. The Role of Short-Chain Fatty Acids in Health and Disease. Adv Immunol (2014) 121:91–119. doi: 10.1016/b978-0-12-800100-4.00003-9 24388214

[97] Han A Bennett N MacDonald A Johnstone M Whelan J Donohoe DR. Cellular Metabolism and Dose Reveal Carnitine-Dependent and -Independent Mechanisms of Butyrate Oxidation in Colorectal Cancer Cells. J Cell Physiol (2016) 231(8):1804–13. doi: 10.1002/jcp.25287 26661480

[98] Kim S Kim JH Park BO Kwak YS. Perspectives on the Therapeutic Potential of Short-Chain Fatty Acid Receptors. BMB Rep (2014) 47(3):173–8. doi: 10.5483/bmbrep.2014.47.3.272 PMC4163876 24499669

[99] Pan P Oshima K Huang YW Agle KA Drobyski WR Chen X. Loss of FFAR2 Promotes Colon Cancer by Epigenetic Dysregulation of Inflammation Suppressors. Int J Cancer (2018) 143(4):886–96. doi: 10.1002/ijc.31366 PMC6041131 29524208

[100] Pingitore A Gonzalez-Abuin N Ruz-Maldonado I Huang GC Frost G Persaud SJ. Short Chain Fatty Acids Stimulate Insulin Secretion and Reduce Apoptosis in Mouse and Human Islets *In Vitro*: Role of Free Fatty Acid Receptor 2. Diabetes Obes Metab (2019) 21(2):330–9. doi: 10.1111/dom.13529 30203438

[101] Smith PM Howitt MR Panikov N Michaud M Gallini CA Bohlooly YM. The Microbial Metabolites, Short-Chain Fatty Acids, Regulate Colonic Treg Cell Homeostasis. Science (2013) 341(6145):569–73. doi: 10.1126/science.1241165 PMC3807819 23828891

[102] Jung JK Johnson BR Duong T Decaire M Uy J Gharbaoui T. Analogues of Acifran: Agonists of the High and Low Affinity Niacin Receptors, GPR109a and GPR109b. J Med Chem (2007) 50(7):1445–8. doi: 10.1021/jm070022x 17358052

[103] Elangovan S Pathania R Ramachandran S Ananth S Padia RN Lan L. The Niacin/Butyrate Receptor GPR109A Suppresses Mammary Tumorigenesis by Inhibiting Cell Survival. Cancer Res (2014) 74(4):1166–78. doi: 10.1158/0008-5472.Can-13-1451 PMC3944627 24371223

[104] Thangaraju M Cresci GA Liu K Ananth S Gnanaprakasam JP Browning DD. GPR109A Is a G-Protein-Coupled Receptor for the Bacterial Fermentation Product Butyrate and Functions as a Tumor Suppressor in Colon. Cancer Res (2009) 69(7):2826–32. doi: 10.1158/0008-5472.Can-08-4466 PMC3747834 19276343

[105] Singh N Gurav A Sivaprakasam S Brady E Padia R Shi H. Activation of Gpr109a, Receptor for Niacin and the Commensal Metabolite Butyrate, Suppresses Colonic Inflammation and Carcinogenesis. Immunity (2014) 40(1):128–39. doi: 10.1016/j.immuni.2013.12.007 PMC4305274 24412617

[106] Li M van Esch B Henricks PAJ Folkerts G Garssen J. The Anti-Inflammatory Effects of Short Chain Fatty Acids on Lipopolysaccharide- or Tumor Necrosis Factor α-Stimulated Endothelial Cells *via* Activation of GPR41/43 and Inhibition of HDACs. Front Pharmacol (2018) 9:533. doi: 10.3389/fphar.2018.00533 29875665 PMC5974203

[107] Bolden JE Peart MJ Johnstone RW. Anticancer Activities of Histone Deacetylase Inhibitors. Nat Rev Drug Discov (2006) 5(9):769–84. doi: 10.1038/nrd2133 16955068

[108] Lin KT Wang YW Chen CT Ho CM Su WH Jou YS. HDAC Inhibitors Augmented Cell Migration and Metastasis Through Induction of PKCs Leading to Identification of Low Toxicity Modalities for Combination Cancer Therapy. Clin Cancer Res (2012) 18(17):4691–701. doi: 10.1158/1078-0432.Ccr-12-0633 22811583

[109] Xu Z Tao J Chen P Chen L Sharma S Wang G. Sodium Butyrate Inhibits Colorectal Cancer Cell Migration by Downregulating Bmi-1 Through Enhanced miR-200c Expression. Mol Nutr Food Res (2018) 62(6):e1700844. doi: 10.1002/mnfr.201700844 29418071

[110] Kankaanranta H Janka-Junttila M Ilmarinen-Salo P Ito K Jalonen U Ito M. Histone Deacetylase Inhibitors Induce Apoptosis in Human Eosinophils and Neutrophils. J Inflamm (Lond) (2010) 7:9. doi: 10.1186/1476-9255-7-9 20181093 PMC2841159

[111] Aoyama M Kotani J Usami M. Butyrate and Propionate Induced Activated or non-Activated Neutrophil Apoptosis *via* HDAC Inhibitor Activity But Without Activating GPR-41/GPR-43 Pathways. Nutrition (2010) 26(6):653–61. doi: 10.1016/j.nut.2009.07.006 20004081

[112] Schilderink R Verseijden C de Jonge WJ. Dietary Inhibitors of Histone Deacetylases in Intestinal Immunity and Homeostasis. Front Immunol (2013) 4:226. doi: 10.3389/fimmu.2013.00226 23914191 PMC3730085

[113] Cousens LS Gallwitz D Alberts BM. Different Accessibilities in Chromatin to Histone Acetylase. J Biol Chem (1979) 254(5):1716–23. doi: 10.1016/S0021-9258(17)37831-6 762168

[114] de Zoeten EF Wang L Sai H Dillmann WH Hancock WW. Inhibition of HDAC9 Increases T Regulatory Cell Function and Prevents Colitis in Mice. Gastroenterology (2010) 138(2):583–94. doi: 10.1053/j.gastro.2009.10.037 PMC3369426 19879272

[115] Lee SM Donaldson GP Mikulski Z Boyajian S Ley K Mazmanian SK. Bacterial Colonization Factors Control Specificity and Stability of the Gut Microbiota. Nature (2013) 501(7467):426–9. doi: 10.1038/nature12447 PMC3893107 23955152

[116] Jin B Sun T Yu XH Yang YX Yeo AE. The Effects of TLR Activation on T-Cell Development and Differentiation. Clin Dev Immunol (2012) 2012:836485. doi: 10.1155/2012/836485 22737174 PMC3376488

[117] Round JL Mazmanian SK. Inducible Foxp3+ Regulatory T-Cell Development by a Commensal Bacterium of the Intestinal Microbiota. Proc Natl Acad Sci U S A (2010) 107(27):12204–9. doi: 10.1073/pnas.0909122107 PMC2901479 20566854

[118] Telesford KM Yan W Ochoa-Reparaz J Pant A Kircher C Christy MA. A Commensal Symbiotic Factor Derived From Bacteroides Fragilis Promotes Human CD39(+)Foxp3(+) T Cells and Treg Function. Gut Microbes (2015) 6(4):234–42. doi: 10.1080/19490976.2015.1056973 PMC4615798 26230152

[119] Chu H Khosravi A Kusumawardhani IP Kwon AH Vasconcelos AC Cunha LD. Gene-Microbiota Interactions Contribute to the Pathogenesis of Inflammatory Bowel Disease. Science (2016) 352(6289):1116–20. doi: 10.1126/science.aad9948 PMC4996125 27230380

[120] Tang C Kakuta S Shimizu K Kadoki M Kamiya T Shimazu T. Suppression of IL-17F, But Not of IL-17A, Provides Protection Against Colitis by Inducing T(reg) Cells Through Modification of the Intestinal Microbiota. Nat Immunol (2018) 19(7):755–65. doi: 10.1038/s41590-018-0134-y 29915298

[121] D’Angelo M Billings PC Pacifici M Leboy PS Kirsch T. Authentic Matrix Vesicles Contain Active Metalloproteases (MMP). A Role for Matrix Vesicle-Associated MMP-13 in Activation of Transforming Growth Factor-Beta. J Biol Chem (2001) 276(14):11347–53. doi: 10.1074/jbc.M009725200 11145962

[122] Atarashi K Tanoue T Oshima K Suda W Nagano Y Nishikawa H. Treg Induction by a Rationally Selected Mixture of Clostridia Strains From the Human Microbiota. Nature (2013) 500(7461):232–6. doi: 10.1038/nature12331 23842501

[123] Klaasen HL Koopman JP Poelma FG Beynen AC. Intestinal, Segmented, Filamentous Bacteria. FEMS Microbiol Rev (1992) 8(3-4):165–80. doi: 10.1111/j.1574-6968.1992.tb04986.x 1515159

[124] Atarashi K Tanoue T Ando M Kamada N Nagano Y Narushima S. Th17 Cell Induction by Adhesion of Microbes to Intestinal Epithelial Cells. Cell (2015) 163(2):367–80. doi: 10.1016/j.cell.2015.08.058 PMC4765954 26411289

[125] Sano T Huang W Hall JA Yang Y Chen A Gavzy SJ. An IL-23r/IL-22 Circuit Regulates Epithelial Serum Amyloid A to Promote Local Effector Th17 Responses. Cell (2015) 163(2):381–93. doi: 10.1016/j.cell.2015.08.061 PMC4621768 26411290

[126] Chen YR Zhou LZ Fang ST Long HY Chen JY Zhang GX. Isolation of Desulfovibrio Spp. From Human Gut Microbiota Using a Next-Generation Sequencing Directed Culture Method. Lett Appl Microbiol (2019) 68(6):553–61. doi: 10.1111/lam.13149 30835854

[127] Figliuolo VR Dos Santos LM Abalo A Nanini H Santos A Brittes NM. Sulfate-Reducing Bacteria Stimulate Gut Immune Responses and Contribute to Inflammation in Experimental Colitis. Life Sci (2017) 189:29–38. doi: 10.1016/j.lfs.2017.09.014 28912045

[128] Sanders ME. Probiotics: Definition, Sources, Selection, and Uses. Clin Infect Dis (2008) 46 Suppl 2:S58–61; discussion S144-51. doi: 10.1086/523341 18181724

[129] Sartor RB. Probiotic Therapy of Intestinal Inflammation and Infections. Curr Opin Gastroenterol (2005) 21(1):44–50. 15687884

[130] Park JS Choi JW Jhun J Kwon JY Lee BI Yang CW. Lactobacillus Acidophilus Improves Intestinal Inflammation in an Acute Colitis Mouse Model by Regulation of Th17 and Treg Cell Balance and Fibrosis Development. J Med Food (2018) 21(3):215–24. doi: 10.1089/jmf.2017.3990 29336663

[131] Kwon HK Kim GC Kim Y Hwang W Jash A Sahoo A. Amelioration of Experimental Autoimmune Encephalomyelitis by Probiotic Mixture is Mediated by a Shift in T Helper Cell Immune Response. Clin Immunol (2013) 146(3):217–27. doi: 10.1016/j.clim.2013.01.001 23416238

[132] Shin JH Chung MJ Seo JG. A Multistrain Probiotic Formulation Attenuates Skin Symptoms of Atopic Dermatitis in a Mouse Model Through the Generation of CD4(+)Foxp3(+) T Cells. Food Nutr Res (2016) 60:32550. doi: 10.3402/fnr.v60.32550 27802847 PMC5090133

[133] Chae CS Kwon HK Hwang JS Kim JE Im SH. Prophylactic Effect of Probiotics on the Development of Experimental Autoimmune Myasthenia Gravis. PloS One (2012) 7(12):e52119. doi: 10.1371/journal.pone.0052119 23284891 PMC3527378

[134] Rinaldi E Consonni A Cordiglieri C Sacco G Crasà C Fontana A. Therapeutic Effect of Bifidobacterium Administration on Experimental Autoimmune Myasthenia Gravis in Lewis Rats. Front Immunol (2019) 10:2949. doi: 10.3389/fimmu.2019.02949 31956324 PMC6951413

[135] Ruiz L Delgado S Ruas-Madiedo P Sánchez B Margolles A. Bifidobacteria and Their Molecular Communication With the Immune System. Front Microbiol (2017) 8:2345. doi: 10.3389/fmicb.2017.02345 29255450 PMC5722804

[136] Cammarota G Ianiro G Tilg H Rajilić-Stojanović M Kump P Satokari R. European Consensus Conference on Faecal Microbiota Transplantation in Clinical Practice. Gut (2017) 66(4):569–80. doi: 10.1136/gutjnl-2016-313017 PMC5529972 28087657

[137] Sun MF Zhu YL Zhou ZL Jia XB Xu YD Yang Q. Neuroprotective Effects of Fecal Microbiota Transplantation on MPTP-Induced Parkinson’s Disease Mice: Gut Microbiota, Glial Reaction and TLR4/TNF-α Signaling Pathway. Brain Behav Immun (2018) 70:48–60. doi: 10.1016/j.bbi.2018.02.005 29471030

[138] Sun J Xu J Ling Y Wang F Gong T Yang C. Fecal Microbiota Transplantation Alleviated Alzheimer’s Disease-Like Pathogenesis in APP/PS1 Transgenic Mice. Transl Psychiatry (2019) 9(1):189. doi: 10.1038/s41398-019-0525-3 31383855 PMC6683152

